# An exosome-based specific transcriptomic signature for profiling regulation patterns and modifying tumor immune microenvironment infiltration in triple-negative breast cancer

**DOI:** 10.3389/fimmu.2023.1295558

**Published:** 2023-12-06

**Authors:** Han Wang, Ruo Wang, Lei Luo, Jin Hong, Xiaosong Chen, Kunwei Shen, Yang Wang, Renhong Huang, Zheng Wang

**Affiliations:** ^1^ Department of General Surgery, Comprehensive Breast Health Center, Ruijin Hospital, Shanghai Jiao Tong University School of Medicine, Shanghai, China; ^2^ Institute of Microsurgery on Extremities, Department of Orthopedic Surgery, Shanghai Sixth People’s Hospital Affiliated to Shanghai Jiao Tong University School of Medicine, Shanghai, China; ^3^ School of Biomedical Engineering, Shanghai Jiao Tong University, Shanghai, China

**Keywords:** exosome, gene signature, triple-negative breast cancer, immune infiltration, CLDN7

## Abstract

Triple-negative breast cancer (TNBC) is a highly heterogeneous tumor that lacks effective treatment and has a poor prognosis. Exosomes carry abundant genomic information and have a significant role in tumorigenesis, metastasis, and drug resistance. However, further exploration is needed to investigate the relationship between exosome-related genes and the heterogeneity and tumor immune microenvironment of TNBC. Based on the exosome-related gene sets, multiple machine learning algorithms, such as Cox boost, were used to screen the risk score model with the highest C-index. A 9-gene risk score model was constructed, and the TNBC population was divided into high- and low-risk groups. The effectiveness of this model was verified in multiple datasets. Compared with the low-risk group, the high-risk group exhibited a poorer prognosis, which may be related to lower levels of immune infiltration and immune response rates. The gene mutation profiles and drug sensitivity of the two groups were also compared. By screening for genes with the most prognostic value, the hub gene, CLDN7, was identified, and thus, its potential role in predicting prognosis, as well as providing ideas for the clinical diagnosis, treatment, and risk assessment of TNBC, was also discussed. This study demonstrates that exosome-related genes can be used for risk stratification in TNBC, identifying patients with a worse prognosis. The high-risk group exhibited a poorer prognosis and required more aggressive treatment strategies. Analysis of the genomic information in patient exosomes may help to develop personalized treatment decisions and improve their prognosis. CLDN7 has potential value in prognostic prediction in the TNBC population.

## 1 Introduction

Breast cancer is one of the most common causes of cancer-related death in women worldwide (1, 2). TNBC accounts for approximately 15%-20% of all types of breast cancer. There is widespread concern in academia that metastasis is one of the manifesting features of TNBC, and it develops into an uncontrollable phase due to its inherently aggressive clinical behavior and the lack of efficient molecular targets. Despite TNBC patients having unfavorable prognoses, numerous drugs and combination chemotherapy regimens have been developed to improve the survival of these patients (3). Accordingly, a better understanding of tumor budding aids in the development of novel approaches and therapeutics to improve the clinical benefits for TNBC.

Tumor cells are frequently exposed to the tumor microenvironment (TME), which is surrounded by multiple components. Based on sophisticated transcriptomic or genomic sequencing techniques, we attempted to establish the molecular heterogeneity and classifications of TNBC to find more targets for the treatment of this disease. Within the TME, exosomes play an indispensable role in the regulation of tumor progression in TNBC. Exosomes are extracellular vehicles (EVs) with a diameter of approximately 40-160 nm that are secreted by almost all cells (4, 5). Exosomes can include many cellular components, including nucleic acids, lipids, proteins, etc., which can provide rich omics information, such as cell genome, transcriptome, proteome, and metabolome information (6, 7). Increasing evidence has indicated that exosomes are closely related to tumor formation, growth and metastasis and the acquisition of drug resistance (8, 9). Additionally, the regulatory function of exosomes in tumors is related to nucleic acids and is mediated by the horizontal transfer of proteins (10, 11). It is particularly noteworthy that blood-derived exosomes are very easy to sample in a noninvasive way compared to other sampling methods, which makes their clinical application attractive (12). Therefore, further research on the nucleic acids and proteins of breast cancer exosomes is becoming a potential method for the diagnosis and treatment of breast cancer.

Thus, in the present study, we constructed a classification model based on an exosome-specific transcriptomic signature for TNBC to investigate its potential prognostic value. According to the risk score system based on exome-derived signatures, we classified high-risk and low-risk groups of the classification model and then compared the prognostic values, molecular and biological features, gene enrichment pathways, genomic mutation landscape, immune cell infiltration, and drug sensitivity between the two groups. Finally, we integrated and screened a hub gene that was most relevant to prognosis. All these aims are to find optimal treatment strategies for patients with TNBC.

## 2 Methods and materials

### 2.1 TNBC dataset enrollment and preprocessing

Breast cancer patients were separated into two groups. The TNBC group comprises samples from two cohorts: GSE135565 and TCGA-TNBC. Due to the limited sample size of GSE135565 and the specific requirements of machine learning algorithms, we randomly divided the TCGA-TNBC samples into training and testing cohorts using the R package caret The sample size of training cohort is 85, sample size of testing cohort is 36 and sample size of GEO cohort is 83. This partitioning allowed us to construct a robust prognostic model for TNBC. To ensure consistency in data analysis, the RNA-seq raw read count from the TCGA database was converted to transcripts per kilobase million (TPM) and subsequently log-2 transformed. The data obtained from the GEO database were sourced from the Affymetrix^®^ GPL570 platform (Human Genome U133 Plus 2.0 Array). To process the raw data from Affymetrix^®^, we employed the robust multiarray averaging (RMA) algorithm implemented in the Affy package. Moreover, we reannotated the probe sets of the GPL570 array for genes by mapping all probes to the human genome (hg38) using SeqMap.

### 2.2 Construction of a risk score system based on exosome-related signatures

Since this study aims to construct a noninvasive risk score system for TNBC, we used signatures from the ExoCarta database, which contains numerous signatures from human exosomes. We first compared the differentially expressed genes from ExoCarta and investigated the prognostic impact of those genes. In addition, the biological role and mutation pattern of those genes were also analyzed. Next, we constructed the risk score based on integrative analysis containing various algorithms. In general, 87 combinations of 9 machine learning algorithms, including Lasso, Ridge, stepwise Cox, CoxBoost, random survival forest (RSF), elastic network (Enet), partial least squares regression for Cox (plsRcox), supervised principal components (SuperPC), and survival support vector machine (survival-SVM) based on 10-fold cross-validation, were further used to screen out the most valuable signature with the highest C-index. The detailed parameters for machine learning were gamma.mu = 1, opt.meth = “quadprog”, and kernel = “rbf_kernel”.

### 2.3 Multiomics analysis of risk-related genes across cancers

Nine genes of the risk score system were investigated in across cancers at different omics levels, including expression, methylation, mutation, copy number variation (CNV), single-nucleotide variation (SNV), and drug sensitivity analysis. The detailed parameters for this part can be found in previous works (13).

### 2.4 The biological role of the risk score in TNBC

To elucidate the potential biological significance of the risk score in TNBC, we first investigated the differentially expressed genes (DEGs) between the high- and low-risk groups based on the optimal cut-off point. To gain a comprehensive understanding of DEGs, we performed various analyses, including Gene Ontology (GO) analysis, Kyoto Encyclopedia of Genes and Genomes (KEGG) analysis, gene set enrichment analysis (GSEA), and gene set variation analysis (GSVA). These analyses were carried out using the R package ClusterProfiler.

### 2.5 Drug sensitivity analysis and immune infiltration analysis

The susceptibility of patients with TNBC to chemotherapy and molecular drugs was assessed using well-established databases, such as the Genomics of Cancer Drug Sensitivity (GDSC) database. To accurately determine the half-maximal inhibitory concentration (IC50) and validate these calculations, we utilized the R package pRRophetic. To delve into further detail, our analysis revealed a positive correlation. This suggests that an elevated risk score is indicative of resistance to the drugs, while a reduced expression level implies a greater sensitivity to the drug. To assess the presence of immune cells in TNBC patients and ascertain the degree of immune cell enrichment or cellular component scores, we employed multiple established algorithms. Our primary objective was to investigate the impact of the risk score on immune infiltration.

### 2.6 Isolation and *in vitro* supplementation of exosomes

Isolation of exosomes was performed as described in a previous study (14). Cells were cultured in DMEM with exosome-free FBS for 48 h. The cultured medium was corrected and centrifuged at 2200 x g for 15 min and 11,000 x g for 35 min. Then, the supernatant was filtered with a 0.22-µm filter. Subsequently, the medium was centrifuged at 110,000 x g for 100 min. Last, the exosomes were resuspended and then centrifuged at 110,000 x g for 100 min and resuspended in 50 μL of PBS. Protein markers of CD63 and TSG101 were measured by Western blotting. MDA-MB-231 and MDA-MB-1315 cells were cultured in medium containing 100 μg/mL exosomes for 48h.

### 2.7 Western blot

Western blotting was performed as described in our previous study (15). In brief, proteins were extracted, and a Thermo BCA Protein Assay (Scientific™ Pierce™, Thermo Fisher Scientific) was used to measure the concentrations of the lysate proteins. Then, the proteins were separated by 10% sodium dodecyl sulfate−polyacrylamide gel electrophoresis (SDS−PAGE) and transferred onto polyvinylidene difluoride membranes (PVDF). Subsequently, the membranes were blocked with 5% nonfat dry milk and incubated with primary antibodies at 4°C overnight (anti-CD63, 25682-1-AP, 1:1000, Proteintech; anti-TSG101, 28283-1-AP, 1:1000, Proteintech). Afterward, the membranes were incubated with goat anti-rabbit IgG heavy and light chain/horseradish peroxidase at room temperature for 1 h. α-Tubulin was used as a loading control for normalization (α-tubulin antibody, 1:1000). Imaging of the membranes was captured by a Luminescent Image Analyzer detection system (Fujifilm, LAS-4000).

### 2.8 RT−PCR

TRIzol reagent (Invitrogen, Carlsbad, CA, USA) was applied to extract total RNA, which was then reverse transcribed by a RevertAid First Strand cDNA Synthesis Kit (Thermo Fisher, USA). Subsequently, qRT−PCR was performed using SYBR Green PCR Master Mix (Thermo Fisher) on an ABI 7300 system. The primers of the CLDN7 sequences were as follows: forward, GGGTGGAGGCATAATTTTCA; reverse, AGTGCACCTCCCAGGATGAC. The relative expression of CLDN7 was quantified by the comparative 2^-ΔΔCt^ method with GAPDH as an internal control.

### 2.9 Wound healing and transwell assay

A scratch wound-healing assay was employed to measure the cell migration rate. Cell migration and invasion abilities were detected using transwell assays, as described in our published study (16).

### 2.10 Immunohistochemistry analysis of tissue microarray

IHC staining of the CLDN7 protein in the TNBC tissue microarray was performed by incubation with human CLDN7 antibody (10118-1-AP, 1:400, Proteintech) overnight. It was then incubated with goat monoclonal antibody against rabbit antibody (111-035-003, JACKSON, 1:1000) for 1 h at room temperature. The immunohistochemical staining of CLDN7 was evaluated and analyzed by two individual pathologists in our hospital, Anqi Li and Miao Ruan, who were fully blinded to the information of these patients. Protein expression was evaluated based on the immunoreactive score (IRS) (17).

### 2.11 Statistical analysis

All data processing, statistical analysis, and visualization were performed using R software (version 4.2.2), employing the ggplot2, ggpubr, and MOVICS packages. To discern between the low-risk and high-risk subgroups, we employed either Student’s t test or the Wilcoxon test. The chi-square test was employed to compare differences in clinical characteristics and inhibitor response. We assessed the impact of the risk score on patient prognosis, specifically overall survival (OS) and progression-free survival (PFS), utilizing the Kaplan−Meier and log-rank tests. A two-tailed p value test was conducted, with statistical significance defined as p < 0.05. Furthermore, for more comprehensive analysis details, readers can refer to our previous works.

## 3 Results

### 3.1 Landscape of exosome-derived signatures

**Figure 1:**
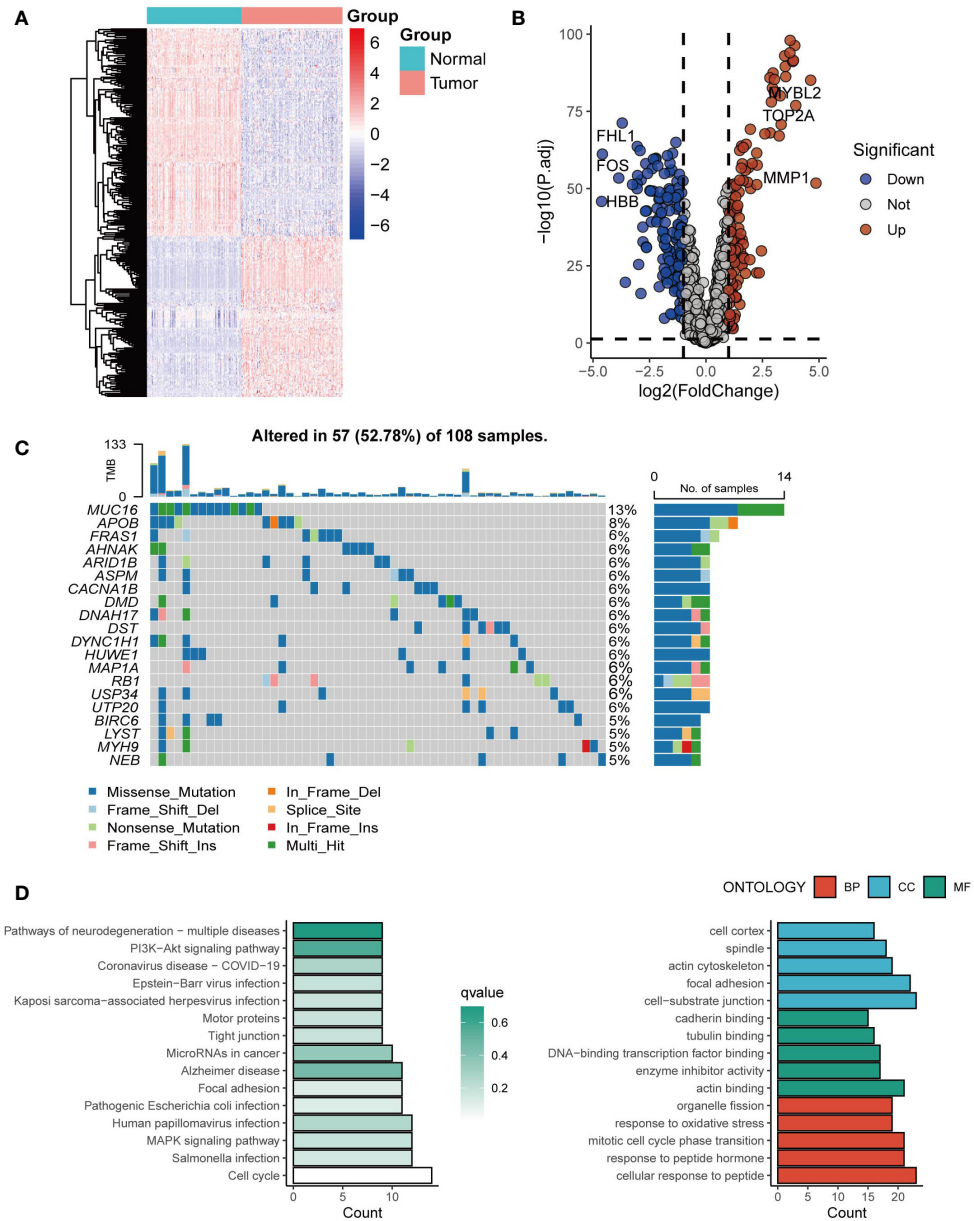
The landscape of exosome-related genes. **(A)** Heatmap of expression profiles of exosome-related genes between cancer tissues and normal tissues. **(B)** The volcano plot showing differentially expressed genes from ExoCarta. **(C)** Waterfall plot showing the mutation patterns of the top 20 most frequently mutated genes. **(D)** Enrichment analysis of the differentially expressed genes.

Exosome-related genes from the ExoCarta database were included, and the expression of the genes in tumor tissues and normal tissues was analyzed. Genes such as MYBL2, TOP2A, and MMP1 were significantly upregulated in tumor tissues (**Figures 1A, B**). The mutation spectrum of the exosome-related gene set is shown in **Figure 1C**, with gene mutations mainly concentrated in MUC16, APOB, and FRAS1 (**Figure 1C**). Further enrichment analysis was conducted on the exosome-related gene set. Genes involved in constructing the risk score model were mainly enriched in pathways of multiple neurogenic diseases and the PI3K Akt signaling pathway (**Figure 1D**).

### 3.2 Construction of a risk score system based on exome-derived signatures for TNBC

**Figure 2:**
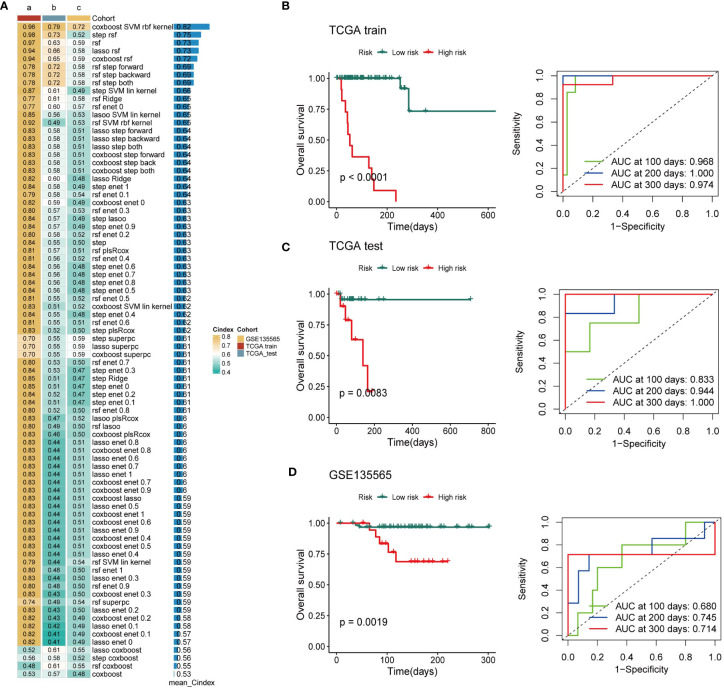
Establishment of a nine-exosome-related gene risk model. **(A)** C-index of each model across the datasets. **(B–D)** Survival analysis for OS and the time-dependent ROC curves for the two risk groups in different cohorts.

Based on the expression profiles of 163 exosome-derived signatures, univariate Cox analysis identified 85 prognostic exosome-related signatures. These genes were subjected to our machine learning-based integrative procedure to develop a consensus exosome-related signature. In the TCGA-TNBC dataset, we fitted 87 kinds of prediction models via the LOOCV framework and further calculated the C-index of each model across all validation datasets. Interestingly, the optimal model was the application of CoxBoost with the highest average C-index (0.82), and this combination model had a leading C-index in the test and independent datasets of TNBC (**Figure 2A**).

Next, a risk score for each patient was calculated using the expression of 9 exosome-related signatures. All patients were assigned into high- and low-risk groups according to the optimal cut-off point determined by the survminer package. To assess the efficacy of the risk score, case data from TCGA-TNBC and GSE135565 were included. OS was analyzed separately for the high-risk group and the low-risk group using Kaplan−Meier curves, and ROC curves at different time points are presented (**Figures 2B–D**). There was a significant difference in OS between the high-risk group and the low-risk group. The AUC values in different databases indicate that this risk score has promising prognostic predictive efficacy.

### 3.3 Landscape of nine risk score system genes at the multiomics level across cancers

To further investigate the 9 genes involved in constructing the risk score model, we analyzed their expression patterns across cancers. The results showed that the 9 genes were expressed to different degrees across cancers (**Figure S1A**). The 9 genes also have distinct roles in the prognosis of different cancers. For example, MAD2L2 and LRRC61 are risk factors in most cancers, while PFKFB3, ESAM, SYNM, and LRFN5 act as protective factors in most cancers (**Figure S1B**). To further explore the reasons for the differential expression of the 9 exosome-associated genes, we assessed the correlation of genes with CNV across cancers. Most of the gene expression levels were positively correlated with CNV (**Figure S1C**). Heterozygous amplifications were frequently detected in LRRC61, and heterozygous deletions were frequently detected in CLDN7, MAD2L2, and ESAM (**Figure S1D**). SNV analysis showed that the mutation frequency of exosome-associated genes was 100% (n=640) in all of the samples (**Figure S1E**).

### 3.4 Biological processes and pathway activation state in the two groups

**Figure 3:**
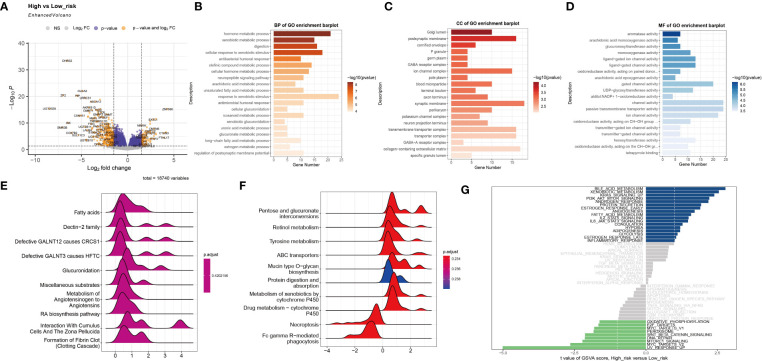
Functional enrichment analysis of the two subtypes. **(A)** The volcano plot shows differentially expressed genes between the high-risk group and the low-risk group. **(B–D)** The GO terms of the BP, CC, and MF categories enriched in the differentially expressed genes. **(E, F)** KEGG analysis of the two subtypes. **(G)** GSVA of the two subtypes.

Based on the established risk score, the high-risk and low-risk groups had different prognoses, and to further assess the biological significance of the risk score, DEGs were analyzed in the two groups (**Figure 3A**). GO enrichment analysis showed that the DEGs were mainly involved in hormone metabolic processes and xenobiotic metabolic processes in the BP category, the Golgi lumen and postsynaptic membrane in the CC category, and aromatase activity and arachidonic acid monooxygenase activity in the MF category (**Figures 3B–D**). The results of KEGG analysis showed that the DEGs are primarily abundant in the Dectin-2 family pathway. Defective GALNT3 promotes the HFTC pathway and the pentose and glucuronate interconversions pathway (**Figures 3E, F**). To further investigate the DEGs, GSVA was performed (**Figure 3G**). The bile acid metabolism, xenobiotic metabolism, and KRAS signaling pathways were significantly upregulated in the low-risk group, while the UV response, MYC targets v2, and MTORC1 signaling pathways were significantly upregulated in the high-risk group.

### 3.5 Immune infiltration and the components of the high- and low-risk groups

**Figure 4:**
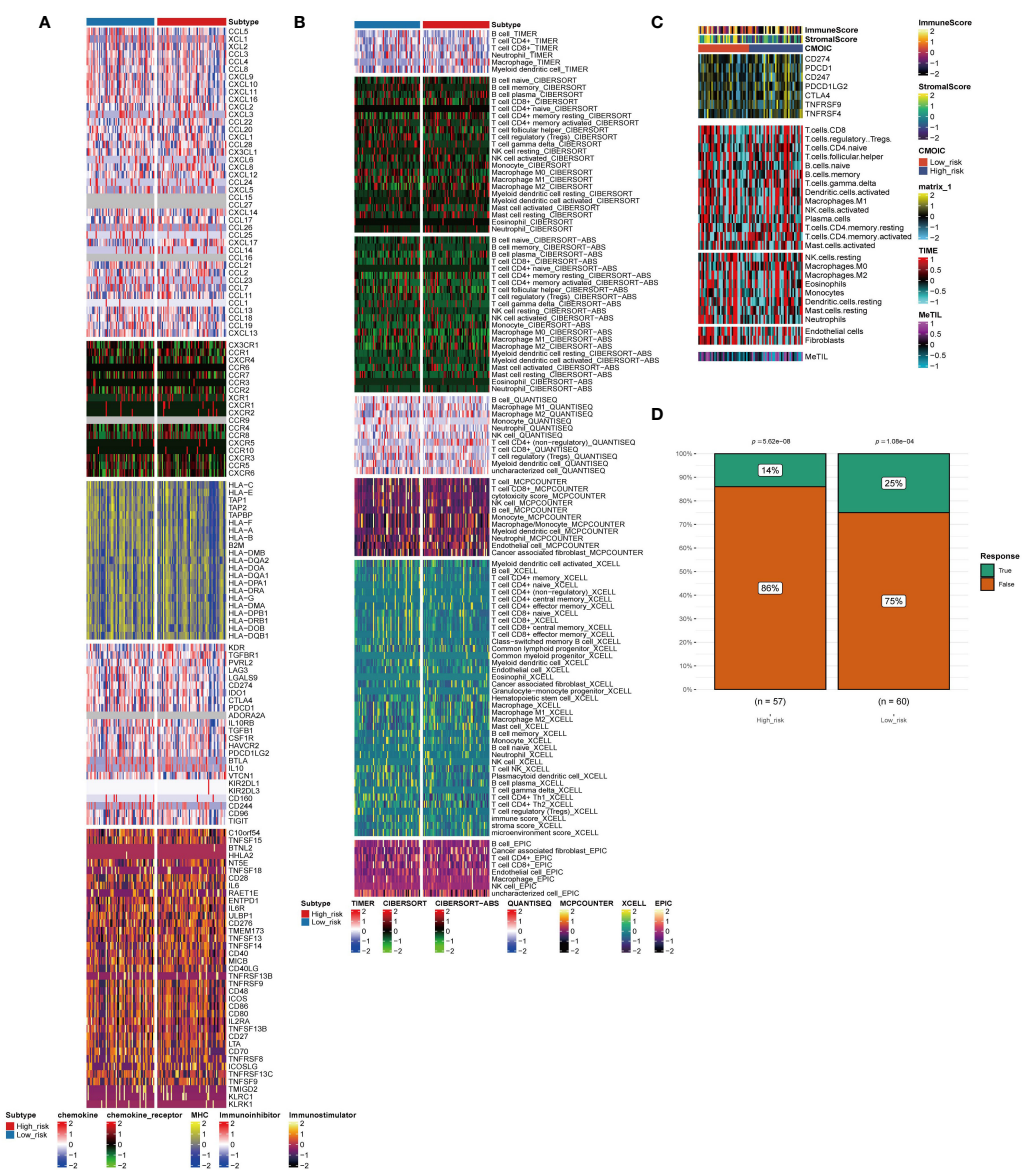
Immune profiling of the two subtypes. **(A–C)** Heatmap indicating the different immune signatures and immune component enrichment between subtypes. **(D)** Differences in the immune therapy response of the high-risk group and low-risk group.

For TNBC, immunotherapy is a valuable treatment strategy. Thus, it is necessary to further analyze the immune heterogeneity between the two groups. The immune-related stimulator signature was analyzed between the two risk groups and the heterogeneity between the two groups in the chemokine, immune-related inhibitor, and other signatures were showed (**Figure 4A**). Next, multiple deconvolution algorithms were used to analyze the immune components of the two groups. The enrichment of different types of macrophages, T cells and B cells differed between high- and low-risk groups (**Figure 4B**). In addition, the low-risk group had higher stromal scores, more neutrophils and fibroblasts infiltration (**Figure 4C**). Consistent with the results above, the low-risk group had a higher response rate to immunotherapy than the high-risk group (25% vs. 14%) (**Figure 4D**).

### 3.6 Mutation landscape of the high- and low-risk groups

**Figure 5:**
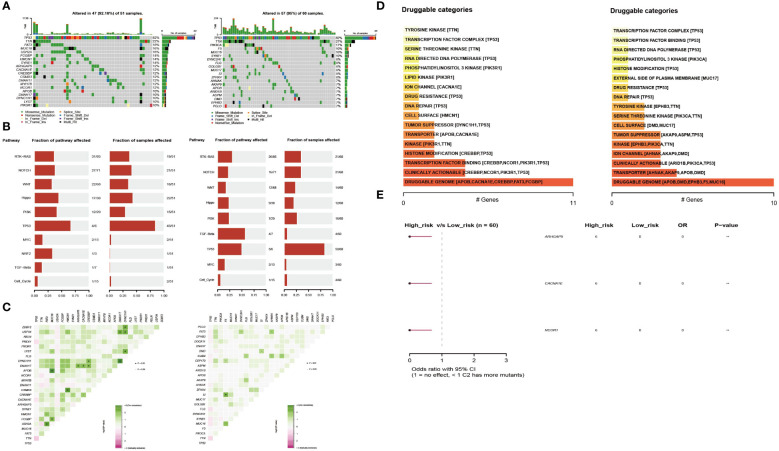
Profiles of somatic mutations and potential targets between the two subtypes. **(A)** Waterfall plot showing the mutation patterns of the top 20 most frequently mutated genes. **(B)** The fraction of pathways or samples of oncogenic signaling pathways in the high-risk group and the low-risk group. **(C)** Co-mutation and existing mutation patterns in the high-risk group and low-risk group. **(D)** Potentially druggable gene categories from the mutation dataset in the high-risk group and the low-risk group. **(E)** Forest plot showing the prognostic impact of mutated signatures in the high-risk group and low-risk group.

To explore the differences between the high- and low-risk groups, we also compared the mutant gene profiles of the two groups. As shown in **Figure 5A**, the overall mutation rate was lower in the high-risk group than in the low-risk group (92.16% vs. 95%). Genes such as MUC16, FAT3, and USH2A had a higher frequency of mutations in the high-risk group (**Figure 5A**). In addition, somatic mutations in classic tumor-associated pathways were compared between the two groups. In the high-risk group, genes with somatic mutations were more concentrated in pathways such as TP53, Hippo, and PI3K, while in the low-risk group, genes with somatic mutations were more concentrated in pathways such as TGF-beta, TP53, and RTK-RAS (**Figure 5B**). Regarding the co-mutation patterns, we found that patterns of APOB-MUC16, DYNC1H1-DNAH17, CSMD3-HMCN1, and USH2A-FAT3 existed in the high-risk group, while the co-mutation of SI-F5 existed in the low-risk group (**Figure 5C**). Druggable genes were classified into two groups according to the potential druggable gene categories, including druggable genome, clinically actionable, transcription factor binding, and transporter (**Figure 5D**). The mutated signatures of ARHGAP5, CACNA1E and NCOR1 play protective roles in the high-risk group (**Figure 5E**).

### 3.7 Drug sensitivity in the high- and low-risk groups

**Figure 6:**
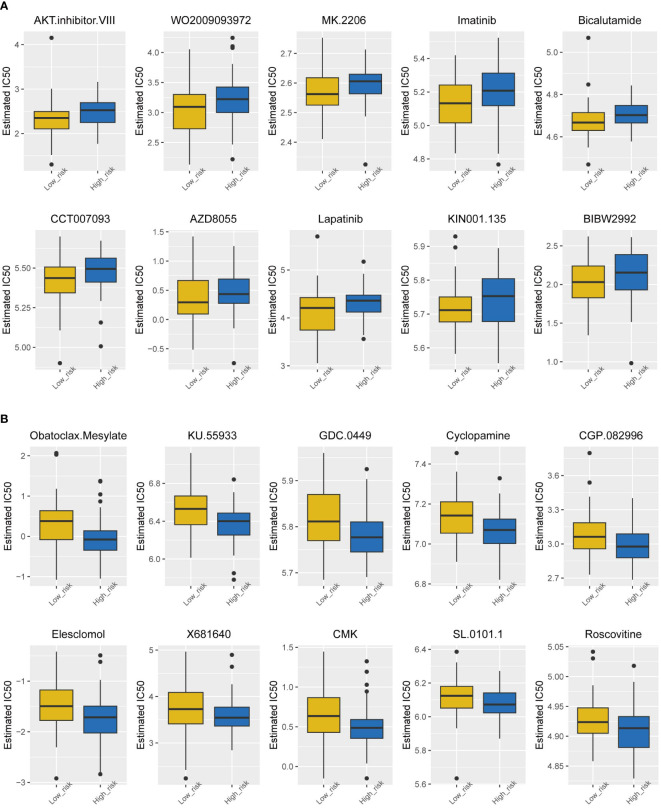
Drug sensitivity analysis of the two subtypes. **(A)** Estimated IC50 of the potential molecular inhibitors of the low-risk group. **(B)** Estimated IC50 of the potential molecular inhibitors of the high-risk group.

Chemotherapy currently has limited efficacy for TNBC, so it is necessary to analyze the sensitivity of drugs in the high-risk group and low-risk group separately, which may lead the development of new drugs. **Figure 6** shows the top 10 sensitive drugs of the two groups by estimated IC50. The low-risk group showed a better response to the AKT inhibitor WO2009093972, MK2206, and imatinib (**Figure 6A**); the high-risk group showed a higher sensitivity to obatoclax mesylate, KU55933, GDC0449, and cyclopamine (**Figure 6B**).

### 3.8 The role of the hub gene CLDN7 in TNBC

**Figure 7:**
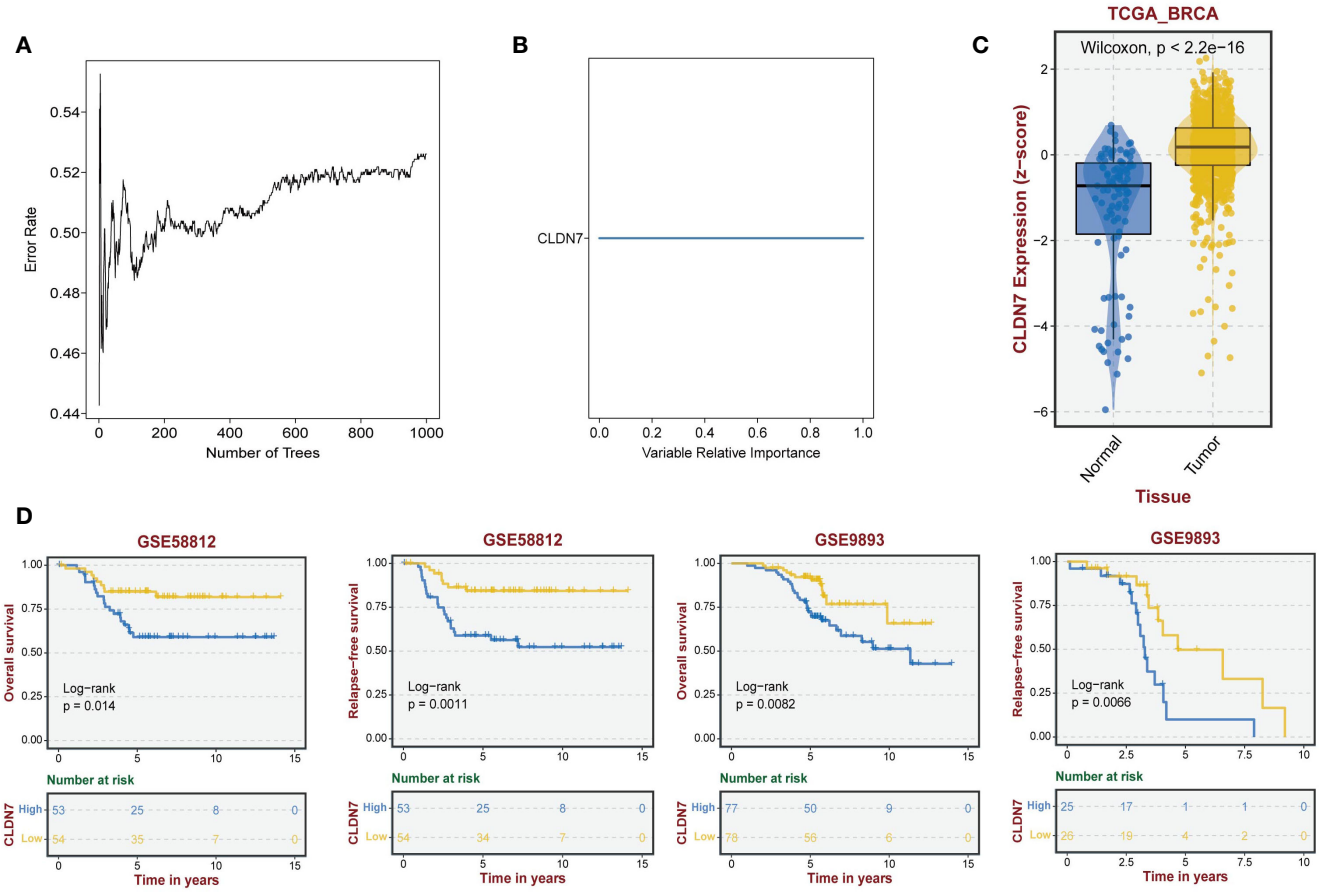
The role of the hub gene CLDN7 in TNBC. **(A, B)** Random forest tree indicating the importance of exosome-related signatures. **(C)** Different expression levels of CLDN7 between normal and tumor tissues. **(D)** The impact of CLDN7 on OS and RFS in TNBC using Kaplan−Meier analysis.

**Figure 8:**
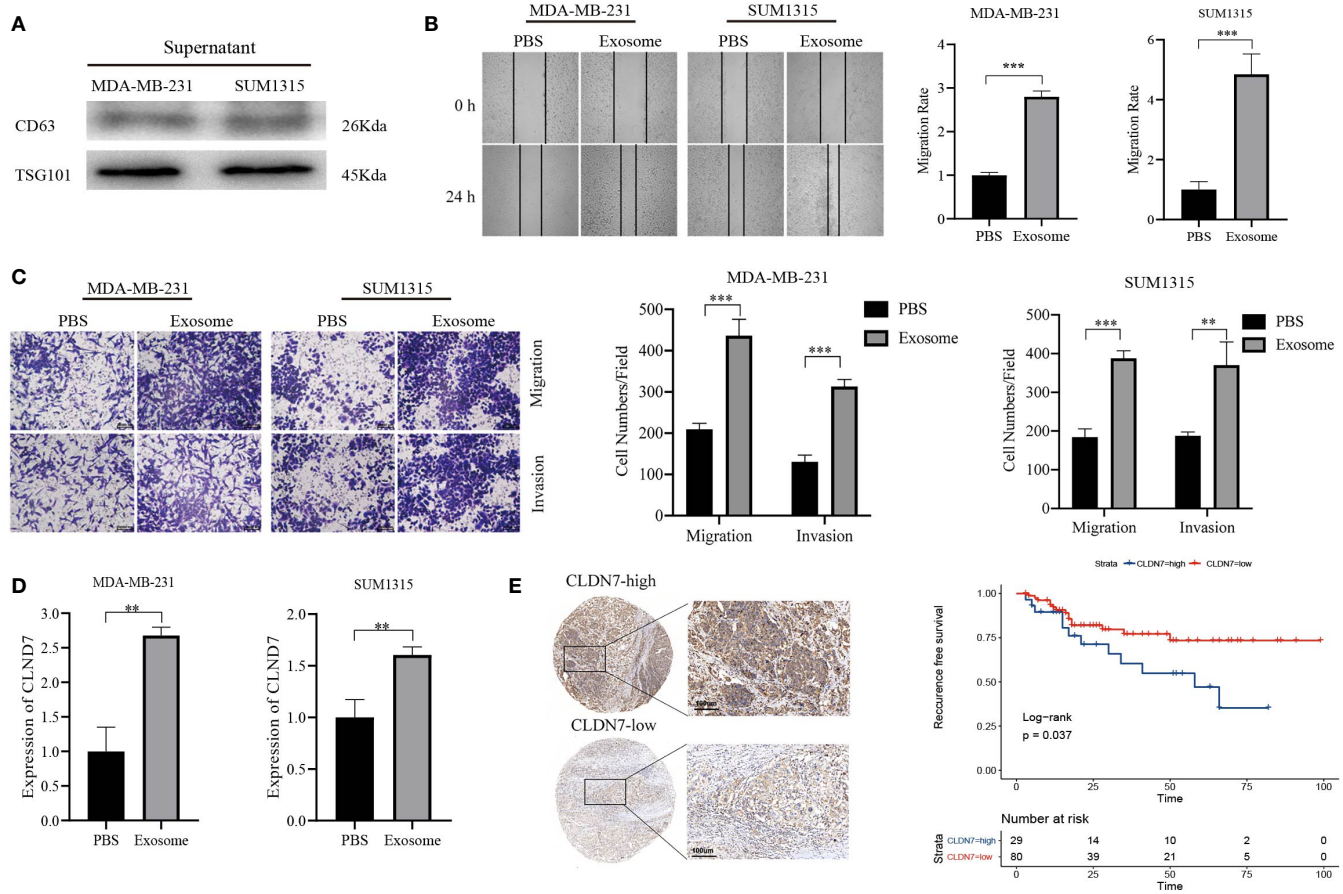
Tumor-promoting effects of CLDN7. **(A)** Western blot of TNBC cell-derived exosomes. **(B)** Wound healing of TNBC cells treated with exosomes. **(C)** Transwell assay of TNBC cells treated with exosomes. **(D)** RT−PCR of TNBC cells after treatment with exosomes. **(E)** Immunohistochemical staining of CLDN7 in TNBC tumor tissue (upper for high-expression group, lower for low-expression group) and survival analysis of different groups of TNBC patients based on IRS. *** *P* < 0.001, ** *P* < 0.01.

Based on the potential of 9 exosome-related signatures in predicting prognosis in TNBC, we wanted to screen for the key gene that is most relevant to prognosis. Using the random forest algorithm, we screened the set of exosome-derived signatures and obtained the gene CLDN7, which may have an important role in TNBC prognosis prediction (**Figures 7A, B**). We included the TNBC population in the TCGA_BRCA dataset to compare the expression of CLDN7 in tumor tissue and normal tissue. CLDN7 was significantly overexpressed in tumor tissue (**Figure 7C**). Then, we explored the prognostic role of CLDN7 in the TNBC population in different datasets, including the GSE58812 and GSE9893 datasets. The OS and PFS of the CLDN7 high-expression group were significantly worse than those of the low-expression group, indicating the potential of CLDN7 as a biomarker for TNBC prognosis (**Figure 7D**). CLDN7 was identified as the hub gene in exosome-derived signatures. Thus, we extracted exosomes from TNBC cell supernatant. Typical exosome markers, such as CD63 and TSG101, were found in cell supernatant exosomes (**Figure 8A**). In addition, we analyzed the biological role of exosomes, and the results demonstrated that exosomes promote TNBC cell migration and invasion (**Figures 8B, C**). Furthermore, we found that CLDN7 was more highly expressed in TNBC cell-derived exosomes (**Figure 8D**). To validate the possible prognostic role of CLDN7, a tissue microarray containing recurrence-free survival (RFS) information of 109 TNBC patients was evaluated. TNBC patients with high CLDN7 expression had poor RFS (**Figure 8E**).

## 4 Discussion

TNBC has the characteristics of high metastasis, high recurrence, and high tumor heterogeneity (18). Therefore, new models that can identify molecular subtypes of TNBC and predict patient prognosis are urgently needed. Increasing evidence suggests that exosomes play an important role in various diseases, including tumors (5, 19). Exosomes are closely related to tumor formation, growth, and metastasis and acquired drug resistance. However, studies on identifying molecular subtypes or constructing prognostic models based on exosomes are still scarce and unknown. In this study, based on the exosome-related gene set, we screened genes associated with patient prognosis and analyzed gene characteristics in terms of biological effects and mutation patterns. A risk score containing multiple algorithms was further constructed. The risk score with the most prognostic predictive value was selected, and the TNBC population was divided into a high-risk group and a low-risk group. The results showed that the prognosis was worse in the high-risk group. We analyzed differentially expressed genes and enriched pathways, immune infiltration landscapes, gene mutations, and drug sensitivity in the two groups.

Although there are multiple mechanisms, including those involving oncogenes and secreted proteins, underlying tumor metastasis of TNBC, the importance of exosomal genes in TNBC tumors has received increasing attention. Exosomes are nanometer-sized extracellular vesicles (EVs) ranging in diameter from 50 to 150 nm. Studies have shown that TNBC exosomes can serve as intercellular messengers to provide intercellular information transduction through the transfer of mRNA in donor exosomes to recipient cells, thereby promoting TME interactions, including immunosuppression and immune escape, vascular generation, genetic information exchange, tumor progression, invasion and metastasis (20–22). For example, Yin-Yuan Mo et al. found that miR-10b can be actively secreted outside the cell through exosomes in metastatic breast cancer MDA-MB-231 cells. Subsequently, exosomal miR-10b could be taken up by different TNBC cells and suppress the protein levels of its target genes, such as HOXD10 and KLF4, thereby promoting breast cancer cell invasion (23). The cisplatin-resistant TNBC cell line established by Chen, Hongfeng, et al. can increase the drug resistance of recipient cells through miR-423-5p secreted by exosomes (24). Interestingly, in addition to the abovementioned effects, exosomes can also directly exclude chemotherapy drugs to form cell drug resistance (25). In addition, in a variety of tumors, such as head and neck squamous cell carcinoma and liver cancer, prognostic models based on exosome-related genes have been established (26, 27). Based on the above studies, TNBC-derived exosomes are considered promising biomarkers for early cancer diagnosis, tumor prognosis, and individualized drug therapy.

For TNBC, an effective drug treatment regimen is critical for clinical treatment. Recently, immune checkpoint inhibitors (ICIs) have received widespread attention due to their excellent efficacy; among these, a checkpoint inhibitor of the anti-PD1 antibody, pembrolizumab, has been approved for advanced PD-L1-positive TNBC (28). However, TNBC patients show strong heterogeneity in response to treatment. For example, in the recently reported Keynote522 study, in early TNBC, both PD-L1-negative and PD-L1-positive patients benefited from pembrolizumab. However, PD-L1 expression could not distinguish responders from non-responders, and subgroup analysis did not provide any effective biomarkers (29). In the recently reported IMPASSION131 study, the combination of paclitaxel and PD-L1 inhibitors failed to improve progression-free survival (PFS) or overall survival (OS) in TNBC patients (30). In addition, chemotherapy is an important treatment modality for TNBC. However, the chemotherapy insensitivity of TNBC may lead to failure of clinical treatment. Previous studies have indicated that exosomes may affect the sensitivity and drug resistance of TNBC to chemotherapy drugs and affect prognosis in various ways (31, 32). Based on this, predicting the efficacy and prognosis of chemotherapy or immunotherapy for TNBC patients has become a key issue. In our model, we found that the high-risk group was more sensitive to obatoclax mesylate, KU55933, GDC0449, and cyclopamine, providing new research directions for the pharmacological treatment of TNBC.

In the risk assessment model we built, we focused on an important hub gene in exosome transcriptomics: CLDN7. Claudin-7 expressed by CLDN7 is an important molecule of tight junctions between cells and maintain cell polarity (33). Increasing evidence shows that the abnormal expression of CLDN7 leads to the destruction of tight junctions between cells, the loss of cell contact inhibition, and abnormal proliferation, which is closely related to the occurrence and development of various malignant tumors. In addition, the abnormal destruction of CLDN7 function is one of the important mechanisms for malignant tumor cells to break away from the primary cancer tissue and cause distant invasion and metastasis. Frédéric Hollande et al. suggested that in colorectal cancer, Tcf-4 promotes high expression of CLDN7 through Sox-9, and the resulting overexpression of claudin-7 promotes the loss of tumor cell polarization and promotes tumorigenesis (34). In addition, studies by Margot Zoller et al. revealed that in colorectal cancer, high expression of CLDN7 is the key to the formation of the EpCAM, claudin-7, CO-029, and CD44v6 complex, and the co-expression level of the complex is positively correlated with poor disease-free survival time (35). In ovarian cancer, a study by Ben Davidson et al. based on 181 tumor-related tissues suggested that high expression of CLDN7 was associated with poor progression and an independent predictor of survival, suggesting the value of CLDN7 as a prognostic factor in ovarian cancer (36). Patrice J. Morin’s research also indicated that the overexpression of CLDN7 in ovarian cancer can promote tumor invasiveness (37). In addition, CLDN7 is associated with the drug resistance of tumor cells. The study by Byoung-Gie Kim et al. demonstrated that CLDN7 is highly expressed in 2774 and HeyA8 human ovarian cancer cells, and inhibiting CLDN7 significantly enhanced the response of 2774 and HeyA8 cells to cisplatin treatment. This suggests that the high expression of CLDN7 may help tumor drug resistance (38). With breast cancer, however, the situation is different. Binghe Xu et al. performed immunohistochemistry on samples from 173 TNBC patients, and the results suggested that CLDN7 could not be used as an independent prognostic factor for TNBC (39). This may be due to the inherent bias of the immunohistochemical experimental method and deserves further study. In summary, CLDN7 plays a role in tumor invasion. Drug resistance is associated with worse patient prognosis and has the potential to be a prognostic biomarker for TNBC.

However, our study has limitations. First, our main findings were derived through a comprehensive bioinformatics analysis. Further experimental verification is still needed, including the detailed mechanism of how CLDN7 in exosomes interacts and how the downstream signaling pathways are controlled. Second, although drug sensitivities differ between groups, further cell or animal experiments are warranted. Finally, even if we validated the prognostic model, some confounding factors, such as race and region, could not be avoided. More independent datasets are needed to reduce potential bias.

In summary, we constructed a risk score model for TNBC based on the exosome-related gene set, categorizing patients into high- and low-risk groups. Detecting exosome-associated genes in patients may become a predictive approach for TNBC prognosis in the future. CLDN7 may serve as a prognostic biomarker for the TNBC population. Our study contributes to a better understanding of the relationship between exosome-related genes and TNBC prognosis and provides new ideas for the clinical diagnosis and treatment of TNBC.

## Data availability statement

The datasets presented in this study can be found in online repositories. The names of the repository/repositories and accession number(s) can be found in the article/**Supplementary Material**.

## Ethics statement

The studies involving humans were approved by Ethical Committee of Ruijin Hospital, Shanghai Jiao Tong University School of Medicine. The studies were conducted in accordance with the local legislation and institutional requirements. The participants provided their written informed consent to participate in this study. Written informed consent was obtained from the individual(s) for the publication of any potentially identifiable images or data included in this article.

## Author contributions

HW: Writing – original draft, Data curation, Formal Analysis, Investigation, Methodology, Project administration, Software. RW: Writing – original draft, Data curation, Formal Analysis, Investigation, Software. LL: Writing – original draft, Data curation, Methodology. JH: Writing – original draft, Data curation, Formal Analysis. RH: Writing – review & editing, Conceptualization, Supervision, Validation. ZW: Writing – review & editing, Conceptualization, Funding acquisition, Supervision, Validation. XC: Writing – review & editing, Methodology. YW: Writing – review & editing, Methodology. KS: Writing – review & editing, Funding acquisition, Validation.

## Supplementary material

Supplementary Figure 1 Dysregulation and genome alterations of exosome-related genes across cancers. **(A)** The gene expression of exosome-related genes in cancer tissues and normal tissues across cancers. **(B)** The relationship between the expression of exosome-related genes and patient survival. **(C)** Correlation analysis of CNVs with the gene expression of exosome-related genes. **(D)** Heterozygous amplification and deletion of exosome-related genes. **(E)** Waterfall plot showing the mutation frequency and type of exosome-related genes across cancers.
