# Supplementary figures and images for: An exosome-based specific transcriptomic signature for profiling regulation patterns and modifying tumor immune microenvironment infiltration in triple-negative breast cancer
Source: Front Immunol. 2023 Dec 6;14:1295558. doi: 10.3389/fimmu.2023.1295558 (PMC10731294; doi:10.3389/fimmu.2023.1295558)

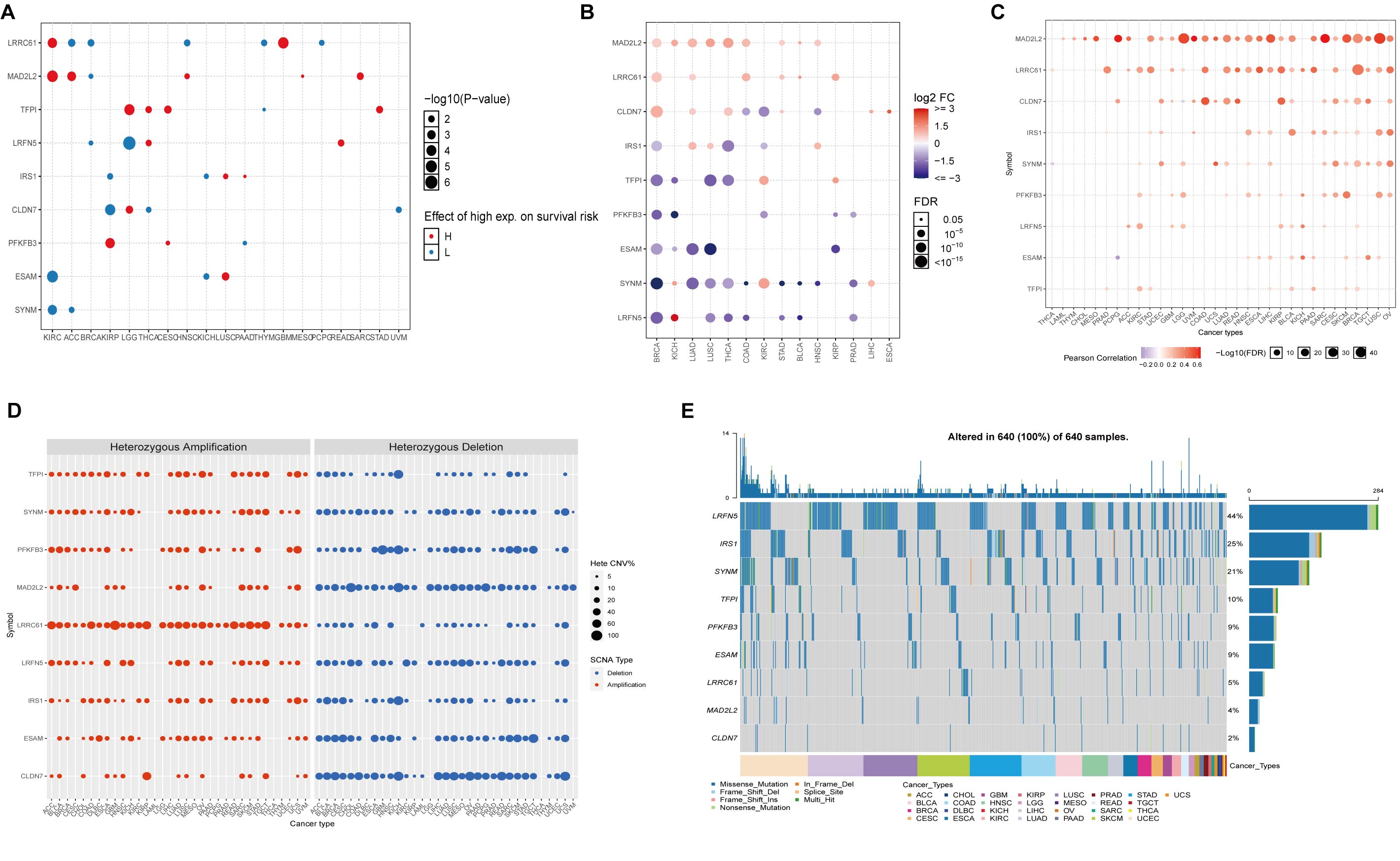

Supplement: Supplementary Figure 1 — Dysregulation and genome alterations of exosome-related genes across cancers. (A) The gene expression of exosome-related genes in cancer tissues and normal tissues across cancers. (B) The relationship between the expression of exosome-related genes and patient survival. (C) Correlation analysis of CNVs with the gene expression of exosome-related genes. (D) Heterozygous amplification and deletion of exosome-related genes. (E) Waterfall plot showing the mutation frequency and type of exosome-related genes across cancers. [file DataSheet_1.zip › FigureS1.jpg]
