# Supplementary material for: An exosome-based specific transcriptomic signature for profiling regulation patterns and modifying tumor immune microenvironment infiltration in triple-negative breast cancer
Source: Front Immunol. 2023 Dec 6;14:1295558. doi: 10.3389/fimmu.2023.1295558 (PMC10731294; doi:10.3389/fimmu.2023.1295558)
Supplement: Supplementary file 2 [file DataSheet_2.docx]

Raw Data can be linked to:

<https://www.jianguoyun.com/p/DYA1iz0Qk9eHDBicvqEFIAA>
